# Comparative Upper Respiratory Tract Transcriptomic Profiling Reveals a Potential Role of Early Activation of Interferon Pathway in Severe COVID-19

**DOI:** 10.3390/v14102182

**Published:** 2022-10-01

**Authors:** Shabir A. Bhat, Tomohiro Shibata, Matthew Leong, Jasmine Plummer, Eric Vail, Zakir Khan

**Affiliations:** 1 Department of Pathology and Laboratory Medicine, Cedars-Sinai Medical Center, Los Angeles, CA 90048, USA; 2 The Center for Bioinformatics and Functional Genomics, Cedars-Sinai Medical Center, Los Angeles, CA 90048, USA; 3 Department of Biomedical Sciences, Cedars-Sinai Medical Center, Los Angeles, CA 90048, USA

**Keywords:** COVID-19, RNA-Seq, IFN-I response, inflammation, viral infection, host-pathogen interactions

## Abstract

Infection with SARS-CoV-2 results in Coronavirus disease 2019 (COVID-19) is known to cause mild to acute respiratory infection and sometimes progress towards respiratory failure and death. The mechanisms driving the progression of the disease and accumulation of high viral load in the lungs without initial symptoms remain elusive. In this study, we evaluated the upper respiratory tract host transcriptional response in COVID-19 patients with mild to severe symptoms and compared it with the control COVID-19 negative group using RNA-sequencing (RNA-Seq). Our results reveal an upregulated early type I interferon response in severe COVID-19 patients as compared to mild or negative COVID-19 patients. Moreover, severely symptomatic patients have pronounced induction of interferon stimulated genes (ISGs), particularly the oligoadenylate synthetase (OAS) family of genes. Our results are in concurrence with other studies depicting the early induction of IFN-I response in severe COVID-19 patients, providing novel insights about the ISGs involved.

## 1. Introduction

With an estimate of over 582 million infections and more than 6.4 million deaths due to severe acute respiratory syndrome coronavirus 2 (SARS-CoV-2) [1], Coronavirus disease 2019 (COVID-19) has upended the lives of 7.7 billion people. The symptoms, including fever, dry cough, and shortness of breath, are common during infection. The response of patients to COVID-19 disease is variable, ranging from complete absence to moderate symptoms [2,3]; however, a small fraction of patients develop severe disease and pneumonia, followed by development of acute respiratory distress syndrome (ARDS) and often times leading to respiratory failure and death [4]. SARS-CoV-2 can infect any person regardless of age or sex and can cause severe disease and death. Since the beginning of this pandemic, understanding the disease pathogenesis has been a focus of COVID-19 research in order to improve the clinical management of this disease. Although the actual cause of severe symptoms is still unclear, early reports suggest a close association between severity of symptoms and dysregulated inflammatory response to viral infection [5,6,7,8,9,10]. Given the extraordinary burden on global human health, understanding the underlying causes of severe disease progression in SARS-CoV-2 infection is crucial in fighting this disease and developing therapeutic strategies. The intense research efforts since the beginning of the pandemic have led to many novel approaches in understanding this disease, which in turn have led to the approval of some emergency pharmacological interventions such as antiviral drugs, anti-SARS-CoV-2 antibodies, convalescent plasma therapy, and other immunomodulatory drugs to prevent this disease [11,12,13]. Despite all these efforts, the cause of respiratory failure in SARS-CoV-2 infection is still unknown. Studies suggest the active involvement of proinflammatory hyperactive immune response is a major underlying cause of severe symptoms and acute lung damage [14].

Immune system activation may act as a double-edged sword, initiating an antiviral defense but also causing lung damage and respiratory failure when hyperactivated [15]. To better define the initial host immune response to SARS-CoV-2, we conducted RNA-Seq analysis on upper respiratory tract samples from patients with a range of clinical symptoms. In this study, we recruited three groups of patients: a negative control group (N-group; *n* = 6), asymptomatic or mildly symptomatic group (A-group; *n* = 6), and severely symptomatic group (S-group; *n* = 5). Nasopharyngeal swab samples were collected at the time of diagnostic confirmation using RT-PCR test. Upon comparing the molecular signatures of host transcriptional response to SARS-CoV-2 infection, certain immune-regulated pathway genes were enriched and activated in severe COVID-19 patients as compared to the other two groups. Our results also indicate a correlation between COVID-19 severity with the initial immune response which may have implications for immunotherapy treatment failure in severe COVID-19 patients.

## 2. Materials and Methods

Patients were tested for COVID-19 using RT-PCR at Cedars Sinai Medical Center and were recruited into this study after being categorized into mildly to moderately symptomatic (*n* = 6), severely symptomatic (*n* = 5) and negative patient groups (*n* = 6). The patient cohort included 9 females and 8 males. Total RNA was isolated from the nasopharyngeal swab samples collected from these patients using RNA extraction kit from Qiagen followed by DNase I treatment. RNA integrity was analyzed with the 2100 Bioanalyzer using the Agilent RNA 6000 Pico or Nano Kit (Agilent Technologies, Santa Clara, CA, USA) and RNA was quantified using the Qubit RNA HS Assay Kit (ThermoFisher Scientific, Waltham, MA, USA). Final libraries were prepared using QIAseq Stranded mRNA Select Kit and concentrations were measured using the Qubit 1X dsDNA HS Assay kit. Finally, libraries were sequenced on NovaSeq 6000 (Illumina, San Diego, CA, USA) at an average sequencing depth of ~10 M reads/sample and 1 × 75 bp sequencing. The demultiplexed raw reads were uploaded to GeneGlobe Data Analysis Center (Qiagen, Germantown, MD, USA) at https://www.qiagen.com/us/resources/geneglobe/, accessed on 1 July 2022 for quality control, alignment, and expression quantification. Briefly, 3′ adapter and low-quality bases were trimmed off from reads first using cutadapt (version 1.13) with default settings, and then reads with less than 16 bp insert sequences or with less than 10 bp UMI sequences were discarded.

After demultiplexing, the sequencing reads were aligned to GRCh38 transcriptome reference containing all the protein coding genes. Gene counts were normalized to FPKM (Fragments per kilo base of transcript per million mapped fragments) values (supplementary Table S1), which were then used for statistical analysis.

## 3. Quantification and Statistical Analysis

Statistical analysis and visualization of RNA-Seq data were performed using GraphPad Prism software v8. Mean, SD, Z-scores, *p*-values and adjusted *p*-values were calculated in Excel. Student’s T-test with Benjamini Hochberg adjusted *p*-values were used for comparison between two groups and significance was evaluated with a threshold of 0.05. The significantly differentially expressed genes were further analyzed after FDR correction with *p* < 0.01 for pathway enrichment analysis using ShinyGo 0.76 software.

## 4. Results and Discussion

RNA-sequencing (RNA-Seq) is a powerful tool to investigate the dynamics of virus–host interactions, allowing for identification and integration of the host transcriptional signatures in predicting the prognosis of infection [5,7,9,16,17]. The variable clinical outcome of the disease ranges from asymptomatic to severe respiratory pneumonia requiring intensive care and ventilator support, suggesting a diverse host response to infection [18,19]. Host response biomarkers are an excellent predictor of disease prognosis and treatment response. Keeping the variability of antiviral host responses in mind, our goal was to understand the host transcriptional response at the early onset of infection. To delineate the mechanism of contrasting variabilities in the clinical outcome of COVID-19 patients, we applied RNA-seq to compare the differences in gene expression profile between asymptomatic, mildly symptomatic, and severely symptomatic patients at the early onset of infection. A total of 17 patients were recruited to be included in this study. The patients were divided into three groups: the SARS-CoV-2 negative group (N-group; *n* = 6), the SARS-CoV-2 positive with absent to mild symptoms group (A-group; *n* = 6), and the SARS-CoV-2 positive with severe symptoms group (S-group; *n* = 5). The demographic characteristics of these patients are mentioned in Table 1. The average age of S-group patients was higher than the other two groups. The patients belonging to A-group were not admitted to the hospital and all recovered from COVID-19. The patients in S-group were all admitted to the hospital and died due to acute respiratory distress syndrome (ARDS) and systemic inflammation.

**Figure 1:**
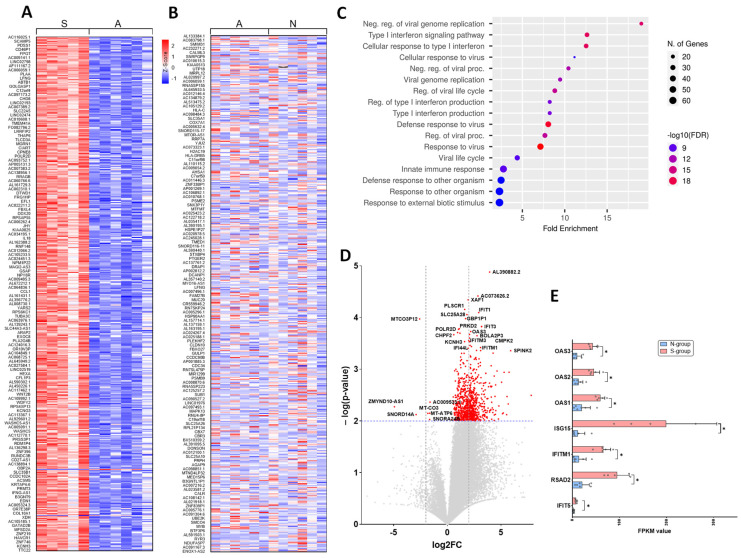
Transcriptomic analysis showing the activation of type I interferon in severe COVID-19. (**A**) Heat map of the transcriptome of patients with severe COVID-19 (S-group) in comparison with mild/asymptomatic COVID-19 (A-group), (**B**) comparison between mild/asymptomatic COVID-19 (A-group) and negative (N-group). (**C**) Bar plot showing the enrichment analysis of genes significantly represented in severe COVID-19 (S-group) as compared to Negative (N-group) samples. (**D**) Volcano plot depicting the significantly (*p*-value < 0.01) differentially upregulated and downregulated genes in severe COVID-19 (S-group) as compared to negative (N-group) patients. (**E**) Bar plot showing the individual interferon stimulated genes upregulated in severe COVID-19 (S-group). * *p* < 0.01.

To understand the differences in the host response to infection, we collected nasopharyngeal swab samples from these patients at the early onset of infection (just after their RT-PCR test came back positive or negative for SARS-CoV-2). We subjected the RNA samples to RNA-Seq analysis and compared the groups among each other. To visualize the differences in the gene expression profiles in this patient cohort, we evaluated significantly differentially expressed genes (*p*-value ≤ 0.01) and plotted them in a heat map representing the statistical Z scores. Heat map visualization plot of the top 500 genes ranked according to the *p*-values shows the contrasting signature in S-group patients’ transcriptome as compared to the A-group and N-group patients’ transcriptome (Figure 1 A). There was no striking difference in the unique transcriptional signature in A-group patients as compared to the N-group control (Figure 1B).

Next, to understand the biological genes/processes that were enriched in the severe COVID-19 data set, we performed gene enrichment analysis of 932 differentially expressed genes sorted according to their *p*-values. The differentially expressed genes with *p*-value ≤ 0.01 were included in our analysis using web based software ShinyGO 0.76 after FDR correction [20]. Out of 932 differentially expressed genes, 100+ genes belonging to type I interferon (INF-I) pathway, particularly interferon-alpha (IFN-α), interferon-beta (IFN-β), and antiviral response pathway, were highly represented in SARS-CoV-2 positive patients as compared to the SARS-CoV-2 negative patients (Figure 1C). Further analysis indicated that major genes upregulated in S-group were related to inflammation. For instance, the interferon stimulated genes (ISGs), including IFIT5, RSAD2, IFITM1, and ISG15 were significantly upregulated (Figure 1D,E, *p* < 0.01). In contrast, we found downregulation of metabolism associated genes such as ZMYND10, SNORD14A, MTND6, MT-CYB in the S-group as compared to the A- and N-group (Figure 1D,E). We also observed the oligoadenylate synthetase (OAS) family of ISGs to be upregulated in the S-group. OASs have been known for their key roles in antiviral defense [21]. OAS proteins sense cytosolic dsRNA, trigger the formation of 2′-5′-linked oligoadenylate (2′-5′A), and activate ribonuclease L (RNaseL) to degrade viral genomes [22]. There are three OAS family proteins (OAS1, OAS2, OAS3) present in humans. A recent report using a CRISPR activation screen identified OAS1 as a major antiviral factor in restricting SARS-CoV-2 infection [23]. Notably, in this CRISPRa screening study [23], OAS2 and OAS3 were not identified to have an antiviral role. However, in our study, we found that all three OAS genes—OAS1, OAS2 and OAS3—were induced in severe COVID-19 samples and may have implications on host response to COVID-19 (Figure 1D,E). More experimental evidence is needed to explore the role of OAS proteins in the development of severe COVID-19 disease.

Previous studies have demonstrated that IFN-I pathway and ISGs are the main pathways in general host response to respiratory viruses [24]. In contrast, unlike other SARS-CoV viruses where IFN-I response is dampened initially [25], our study indicates that the IFN-I response against SARS-CoV-2 starts at the initial encounter with the virus, and this response is significantly stronger in patients who are at a risk of developing severe disease. However, several clinical studies have highlighted that COVID-19 patients with severe disease exhibit suppressed IFN-I response [7,8,10,26]. This contradiction in results between the studies regarding association of IFN-I responses with disease severity in COVID-19 patients can be attributed to different sampling time points, sample types, disease severity, and type of tissue/cells analyzed. Our findings support the concept that early IFN-I response is inversely correlated to disease progression. We postulate that the IFN-I response is high in potentially severe patients at the early encounter with SARS-CoV-2 and leads to immune exhaustion as disease progresses and hence, the decrease in immune response observed in later stages of COVID-19 disease.

The induced inflammatory response in COVID-19 patients, such as induced interleukin-6 (IL-6), CXCL10, and TNF-α, can lead to hyperactivation of Th1/Th17 responses resulting in recruitment and activation of pro-inflammatory neutrophils and macrophages in lungs [27]. This has been proposed as the prime reason for failure to resolve inflammation in severely symptomatic patients [16,28]. Additionally, SARS-CoV-infected mice exhibited induced pro-inflammatory response and cytokine firing, resulting in pneumonia and lung damage through the recruitment of inflammatory monocyte-macrophages (IMMs). Perhaps, due to the higher levels of IFN-I at the initial phase of infection in potentially severe patients, this mechanism may explain progression to severe disease. Indeed, IFN-I receptor knockout in mice was protective against severe viral lung disease [29,30].

**Figure 2:**
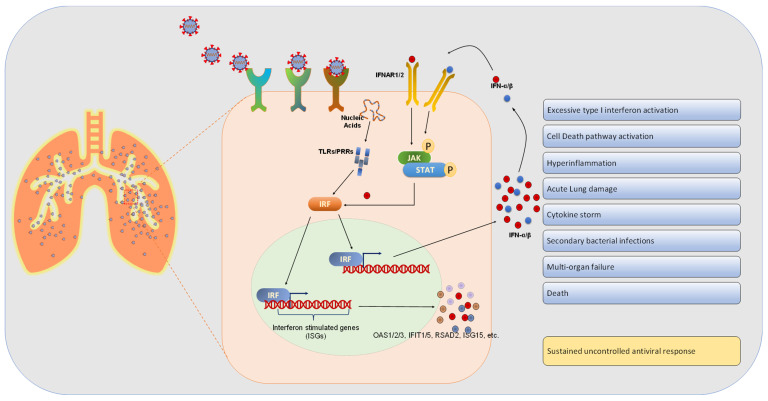
Schematic illustrating the effects of upregulated IFN-I response in hyperinflammation and severe COVID-19 patients supported by transcriptomic analysis.

In conclusion, our study shows a significant difference in the gene expression profile between asymptomatic and severe COVID-19 patients. The severely symptomatic patients exhibited induced IFN-I response as compared to the negative group or asymptomatic positive patients. We hypothesize that the hyperinflammatory type-I response to SARS-CoV-2 infection could further lead to cytokine storm, lung damage, secondary infections, and ultimately multi organ failure (Figure 2). In this study, comorbidities such as hypertension and cardiovascular diseases were not a major issue due to their distribution among all patient cohorts. Future research using larger sample sizes should take caution against the effects of existing comorbidities in patients and treat them as covariables when analyzing correlation in gene expression in severe COVID-19 patients. Although further research must be done, the significant associations found between the early IFN-I response and potential disease severity could serve as biomarkers to predict clinical outcome.

## Figures and Tables

**Table 1:** Demographic Characteristics of COVID-19 patient Cohort.

	Patient ID	Age	Gender	Smoking	Outcome	Comorbidity	Race	Clinical Symptoms at Presentation	Lowest O_2_ Saturation in ED
Negative (N)-Group	Patient 1	55	F	Former	Released	SLE with nephritis, CKD, CVA, seizure disorder, wheelchair bound	Non-Hispanic; Black	Dry cough, malaise, fatigue, chest pressure, mild dyspnea	NA
	Patient 2	37	F	Never	Released	Asthma	Non-Hispanic; White	Shortness of breath, coughing	NA
	Patient 3	71	F	Never	Deceased	PBC cirrhosis s/p liver transplant	Non-Hispanic; White	Febrile neutropenia (concern for GVHD)	NA
	Patient 4	79	M	Former	Released	Ischemic CMY, HTN, HLD, DM, CKD	Non-Hispanic; White	Cough, shortness of breath	NA
	Patient 5	78	F	Former	Released	Dementia	Non-Hispanic; White	Fever, facial swelling	NA
	Patient 6	36	F	Never	Released	Migraines, anxiety	Hispanic; White	Cough, chest tightness	NA
Positive Not Admitted (A)-Group	Patient 7	51	F	Never	Released	None	Non-Hispanic; White	Chest pain, palpitations	99
	Patient 8	43	F	Never	Released	Pregnant, None	Non-Hispanic; Black	Shortness of breath, chills, malaise/fatigue, cough, wheezing	98
	Patient 9	64	M	Former	Released	HLD, HTN	Non-Hispanic; White	Fever, nasal congestion, sore throat	94
	Patient 10	28	M	Never	Released	GAD, Depression, Obesity	Hispanic	Chest pain, coughing, wheezing, heartburn	99
	Patient 11	36	F	Never	Released	None	Non-Hispanic; Asian	Fever, body aches, chills, loss of taste/smell, cough, shortness of breath,	97
	Patient 12	32	M	Never	Released	None	Non-Hispanic	Subjective fever and chills	100
Positive with Severe Symptoms (S)-Group	Patient 13	69	M	Never	Deceased	GAD	Unknown	Chills, fevers, cough, shortness of breath	86 on NRB 15L
	Patient 14	73	F	Never	Deceased	Quadriplegia, DM, large sacral wound present	Non-Hispanic; White	Fever, rhinorrhea, AMS	97
	Patient 15	59	M	Never	Deceased	HTN	Non-Hispanic; Black	Shortness of breath, cough, fevers, abdominal pain	92 on 3L nasal cannula
	Patient 16	47	M	Never	Deceased	None	Non-Hispanic; Black	Fevers, myalgias, fatigue, headache, sore throat, shortness of breath	95
	Patient 17	87	M	Never	Deceased	HTN, Dementia	Non-Hispanic; Black	Weakness, nausea, vomiting	94

SLE: Systemic lupus erythematosus, CKD: Chronic kidney disease, HTN: Hypertension, HLD: Hypersensitivity lung disease, CVA: cerebrovascular accident, CMY: Cardiomyopathy, DM: Diabetes mellitus, GAD: Generalized anxiety disorder, GVHD: Graft-versus-host disease, AMS: altered mental status, NA: not applicable.

## Data Availability

Analyzed data is included in the main text and supplementary files. Raw data can be made available on request from the corresponding author (zakir.khan@csmc.edu).

## Supplementary Materials

The following supporting information can be downloaded at: https://www.mdpi.com/article/10.3390/v14102182/s1.
